# Social Rhythms, Depressive Symptoms, and Quality of Life: An Unbreakable Bond in an Older Adult Sample

**DOI:** 10.3390/ijerph23050583

**Published:** 2026-04-30

**Authors:** Cesar Ivan Aviles Gonzalez, Massimo Tusconi, Sergio Machado, Haidy Rocio Oviedo Cordoba, Doriam Esperanza Camacho Rodriguez, Rober Romero Ramirez, Francesco Pegreffi, Michela Atzeni, Dhurata Ivziku, Marzia Lommi, Vanessa Barrui

**Affiliations:** 1 Department of Medicine and Surgery, University of Kore, Piazza dell’Università, 94100 Enna, Italyfrancesco.pegreffi@unikore.it (F.P.);; 2 Center of Liason Psychiatry and Psychosomatics, University Hospital of Cagliari, 09126 Cagliari, Italy; 3 Center of Neuroscience, Neurodiversity Institute Queimados-RJ, Pacaembu 26325-010, Brazil; secm80@gmail.com; 4 Facuto of Nursing, Universidad del Magdalena, Santa Marta 470001, Colombia; 5 Nursing Faculty, Universidad Cooperativa de Colombia, Bogota 110110, Colombia; 6 Faculty of Administrative, Accounting, and Economic Sciences, Rectorate and Vice-Presidency for Research, Universidad Popular del Cesar, Valledupar 200001, Colombia; roberromero@unicesar.edu.co; 7 Department of Health Professions, Fondazione Policlinico Universitario Campus Bio-Medico, 00128 Rome, Italy; 8 Department of Biomedicine and Prevention, University Tor Vergata, 00133 Rome, Italy; 9 Department of Mental Health and Addiction, ASL Ogliastra, Via Is Piscinas 5, 08045 Lanusei, Italy

**Keywords:** social rhythm dysregulation, depressive symptomatology, quality of life, aging, behavioral synchronization

## Abstract

**Highlights:**

**Public health relevance—How does this work relate to a public health issue?**

- Late-life depression and reduced quality of life are major public health concerns in aging societies; this study links these outcomes to irregular daily social routines (sleep, meals, social contact) in community-dwelling older adults.
- It frames social rhythm dysregulation as a potentially modifiable risk factor associated with both depressive symptoms and poorer health-related quality of life, making it relevant for prevention in community and primary care settings.

**Public health significance—Why is this work of significance to public health?**

- The study reports that greater rhythm dysregulation correlates with more depressive symptoms (BSRS vs. PHQ-9: r = 0.41) and lower quality of life (BSRS vs. SF-12 total: r = −0.39). These are values reported in the Results Section of the article.
- In multivariable analysis, both rhythm dysregulation and depressive symptoms independently predict worse quality of life (reported coefficients: BSRS β = −0.1863; PHQ-9 β = −0.2885; model R^2^ = 0.148), supporting rhythm regularity as a distinct target—not just a by-product of mood.

**Public health implications—What are the key implications or messages for practitioners, policy makers and/or researchers in public health?**

- Combine depression screening (e.g., PHQ-9) with a brief assessment of routine regularity (e.g., BSRS) in older adults, and implement scalable interventions: stable sleep/meal schedules, structured physical activity (often suggested as morning exercise), strengthened social contact, and “light hygiene” (reducing artificial light/screen exposure late in the day).
- Because the design is cross-sectional (no causal inference), priority next steps include longitudinal studies and pragmatic trials using objective measures (e.g., actigraphy/light sensors) to test whether rhythm-stabilizing interventions improve depression and quality-of-life outcomes at the population level.

**Abstract:**

Background: Disruptions in social and circadian rhythms are increasingly recognized as key contributors to depressive symptomatology and impaired quality of life, particularly in older adults, for whom daily regularity represents a crucial determinant of psychological and functional stability. Understanding the interplay between rhythm dysregulation, mood disturbances, and perceived well-being may inform preventive strategies in aging populations. Objective: This study aimed to examine the association between social rhythm dysregulation, depressive symptoms, and perceived quality of life in a sample of community-dwelling older adults. Methods: A cross-sectional observational study was conducted among 119 older adults (mean age 72.26 ± 4.72 years) enrolled in an active aging program. Social rhythms were assessed using the Brief Social Rhythm Scale (BSRS), depressive symptoms with the Patient Health Questionnaire-9 (PHQ-9), and health-related quality of life with the SF-12 Health Survey. Pearson correlation analyses and multiple linear regression models were applied. Results: Greater social rhythm dysregulation was significantly associated with higher depressive symptom levels (r = 0.41, *p* < 0.001) and lower perceived quality of life (r = −0.39, *p* < 0.001). In multivariate analyses, both rhythm dysregulation (β = −0.1863, *p* < 0.001) and depressive symptoms (β = −0.2885, *p* = 0.037) independently predicted poorer quality of life. Conclusions: In community-dwelling older adults, irregular social rhythms and depressive symptoms are independently and jointly associated with reduced quality of life. These findings highlight social rhythm regulation as a relevant and potentially modifiable target for preventive and supportive interventions aimed at promoting mental well-being and resilience in later life.

## 1. Introduction

Social rhythm regularity is increasingly recognized as a behavioral correlate of mental well-being, particularly in later life, when circadian plasticity is reduced and daily routine stability may be especially relevant for psychological and functional adaptation. Previous research suggests that greater disruption of daily social rhythms is associated with depressive symptoms and poorer health outcomes. Against this background, the aim of this study was to examine the association between social rhythm dysregulation, depressive symptoms, and perceived quality of life in a sample of community-dwelling older adults [1].

From a psychosocial perspective, the regularity of social rhythms (e.g., consistent schedules for sleep, meals, and social contact) is robustly associated with fewer depressive and anxiety symptoms. Studies in clinical and nonclinical populations show that greater social rhythmicity is associated with lower affective psychopathology and that this rhythmicity moderates the impact of sleep fragmentation on depressive symptoms; for example, in veterans with PTSD (Post-traumatic stress disorder) and major depression, social rhythm regularity buffered the relationship between sleep disruption and depressive symptoms [2,3]. These findings position social rhythms as a plausible preventative and therapeutic target, beyond the specific symptom “sleep,” along the mental health–illness continuum.

The COVID-19 pandemic offered a “natural experiment” to test these hypotheses. In community-dwelling older adults, the prior preservation of social and behavioral rhythms was associated with greater resilience to pandemic stress and a lower likelihood of depression during lockdowns [4]. Indeed, a low rate of clinically relevant depressive symptoms has been reported in this age group when their rhythms remained stable [5]. There is evidence that pre-pandemic structured physical activity contributed to this resilience, acting as a behavioral chronobiological intervention [6]. Taken together, these results reinforce the idea that maintaining regular daily life patterns can mitigate the risk of depression in older adults, even under extreme stressors.

Genetic variation in circadian clock genes (e.g., BMAL1) has been linked to psychiatric and cardiometabolic traits, supporting a shared circadian basis of mental and physical health [7]. Although the specific causal mechanisms are still under study, these trans-ancestral associations underscore the etiological relevance of the circadian system in psychopathology.

Clinically, sleep disturbances are a hallmark of depression and a modulator of its severity. Beyond insomnia as a symptom, circadian sleep dysfunction has been observed to contribute to systemic inflammatory processes and thus to increased suicidal risk [8]. In bipolar disorder, the presence of circadian rhythm sleep–wake disorders (CRSWDs), particularly delayed phase disorder, predicts a shorter time to affective relapse in euthymic patients [9]. On the other hand, rhythm dysregulation extends to domains such as eating: in major depression, disruption of biological rhythms in eating, activity, and social life is associated with greater severity, and irregularity in eating patterns is related to greater intensity of suicidal ideation [10].

Circadian disruption may influence mood through neuroendocrine mechanisms, including alterations in melatonin and hormonal regulation. Environmental factors such as artificial light at night (ALAN) further perturb circadian rhythms and sleep–wake cycles, with potential consequences for mental and brain health [10,11,12,13,14]. Furthermore, abnormal light exposure may disrupt melatonin and broader hormonal regulation, with downstream effects on metabolic and mental health [11,15].

From an evolutionary perspective, environmental factors such as artificial light and noise may interact with individual vulnerability, potentially influencing energy regulation and mood-related traits [16,17,18].

Within this context, constructs such as the dysregulation of mood, energy, and social rhythms (DYMERS) may offer a useful interpretative framework for understanding the co-occurrence of rhythm disruption and reduced well-being. However, it is important to note that the present study does not directly test this construct, and its inclusion is intended only to provide a broader conceptual perspective [19,20]. The perceived quality of life emerges as a central feature in DYMERS and may serve as a possible marker of the tipping point between adaptive hyperenergy and frank psychopathology, consistent with previous findings on quality of life and habitat (urban versus rural) [21,22,23,24,25,26].

The interface between genetics and the hyperenergetic phenotype adds nuances: variants classically associated with bipolar disorder also appear in individuals without an affective diagnosis but with hyperactivity and novelty-seeking traits, suggesting that certain genetic “risks” could be expressed as non-pathological behavioral traits that, under stressful environments and disorganized rhythms, lead to functional impairment [27,28]. This framework invites more precise phenotyping (e.g., subtypes with rhythmic dysfunction), which could improve risk stratification and guide interventions focused on rhythm stabilization, nighttime light hygiene, and structured physical activity.

Beyond their relevance for mood regulation, social and biological rhythms occupy a central position in contemporary psychiatric models that conceptualize mental disorders along dimensional psychopathological continua rather than categorical boundaries [29,30,31,32]. Increasing evidence suggests that disturbances in daily regularity represent a shared vulnerability substrate across diagnostic spectra, from unipolar depression to bipolar disorders, anxiety conditions, trauma-related syndromes, and even somatic symptom disorders [33,34,35,36]. Within affective psychopathology, rhythm disruption has been proposed not merely as a secondary manifestation but as a potential upstream driver of affective instability, influencing emotional reactivity, reward processing, and stress responsivity. This notion is particularly salient within newer frameworks such as the bipolar spectrum and the DYMERS construct, which highlight how dysregulated energy, sleep–wake patterns, and social behaviors may signal a pre-syndromic or subthreshold state in which individuals retain functional autonomy yet experience a progressive erosion of resilience. Importantly, these rhythm-related vulnerabilities appear to operate through transdiagnostic mechanisms: alterations in the synchronization between central and peripheral clocks may amplify inflammatory activation, modify neurosteroid homeostasis, impair executive control, and dysregulate circadian gating of emotional stimuli. Thus, rhythmicity becomes both a biological integrator and a behavioral marker, bridging mood, cognition, somatic states, and quality of life. In older adults, who exhibit reduced circadian plasticity, cumulative medical comorbidity, and increased sensitivity to environmental zeitgebers, even subtle rhythm fragmentation may precipitate disproportionate psychological and functional consequences. Framed within this broader psychiatric context, the study of social rhythms in aging is not only relevant for understanding depression risk but also for identifying early markers of systemic mental–physical vulnerability, framing rhythm regulation as a cornerstone of preventive psychiatry across the lifespan.

The aim of this study was to examine the association between social rhythm dysregulation, depressive symptoms, and perceived quality of life in a sample of community-dwelling older adults. We hypothesize that (a) greater rhythm dysregulation in sleep, activity, eating patterns, and social contacts will be associated with poorer quality of life and (b) rhythm dysregulation and depressive symptoms will each be associated with lower quality of life, in line with the broader conceptual framework of DYMERS [19,20,21,22]. Evaluating these links in community-dwelling older adults will allow us to identify preventive targets (e.g., rhythm stabilization, light hygiene, exercise) and more precisely profile at-risk subgroups.

While broader chronobiological and transdiagnostic frameworks provide a useful conceptual background, the present study is specifically focused on examining the cross-sectional associations between self-reported social rhythm regularity, depressive symptoms, and perceived quality of life in community-dwelling older adults. Therefore, the findings should be interpreted within the scope of these measured variables and the observational design.

## 2. Materials and Methods

### 2.1. Study Design and Parent Study Context

The present study is a secondary cross-sectional analysis of baseline data collected before the intervention phase of the parent randomized controlled trial, Active Elderly and Health—Can Moderate Exercise Improve Health and Wellbeing in Older Adults? [37]. The parent trial was designed to enroll 120 participants aged 65 years or older, allocated to two groups of similar size, and included baseline, post-treatment, and follow-up assessment [37]. Of the 120 participants planned in the parent trial, 119 were included in the present secondary baseline analysis based on the availability of data for the variables of interest. The present analysis did not use follow-up or intervention data and included baseline participants with available data on the variables examined in this study.

The primary outcome of the present analysis was perceived health-related quality of life, measured with the SF-12. Social rhythm dysregulation was assessed using the Brief Social Rhythms Scale (BSRS), and depressive symptoms were assessed using the PHQ-9 [36,37,38].

### 2.2. Participants and Recruitment

Participants were community-dwelling older adults enrolled in an active aging program. They were recruited voluntarily through an announcement published in the most widely read local newspaper in the area. All participants were from urban settings.

The inclusion criterion for the active aging study was the absence of contraindications to moderate-intensity physical exercise, documented by a medical certificate. Only severe impairment due to severe diseases was considered an exclusion criterion; therefore, participants with mild diabetes or other mild medical conditions were not excluded.

Participants were relatively functionally independent older adults, as eligibility required the absence of contraindications to moderate-intensity physical activity. This should be considered when interpreting the representativeness of the sample.

### 2.3. Measures

#### 2.3.1. Brief Social Rhythms Scale

The Brief Social Rhythms Scale (BSRS) is a validated and simplified Italian version [38] of the Social Rhythm Metric (SRM) [39]. Due to its simplicity, it is suitable for surveys in large samples and for use alongside several other instruments. The BSRS includes 10 items assessing the regularity or irregularity of daily routine activities during the previous week, such as eating, sleeping, and maintaining social contacts. Each item is rated from 1 (maximum regularity) to 6 (maximum irregularity). The BSRS consists of 10 items scored from 1 to 6, yielding a total score range from 10 to 60, with higher scores indicating greater dysregulation of social and behavioral rhythms.

#### 2.3.2. Patient Health Questionnaire-9

To identify depressive symptoms, the Italian version [40] of the 9-item Patient Health Questionnaire (PHQ-9) [41] was used. The PHQ-9 is a self-administered instrument with nine items corresponding to the diagnostic criteria for a depressive episode according to the Diagnostic and Statistical Manual of Mental Disorders [41]. In the present study, only depressive symptom severity was analyzed. The PHQ-9 total score ranges from 0 to 27, with higher scores indicating greater depressive symptom burden.

#### 2.3.3. Short Form-12 Health Survey

To assess perceived health-related quality of life (H-QoL), the 12-item Short Form Health Survey (SF-12) was used [42]. The SF-12 evaluates perceived mental and physical health, functioning, pain, vitality, and possible psychosocial disability or emotional distress [43]. The instrument refers to a broader time frame, approximately the month preceding the interview. Higher SF-12 scores indicate better perceived health-related quality of life [42]. The SF-12 was used as an index of perceived health-related quality of life, with higher scores indicating better perceived health status.

#### 2.3.4. Temporal Framing of the Measures

The BSRS assesses the regularity of daily routines over the previous week, whereas the PHQ-9 captures depressive symptoms typically experienced during the preceding two weeks, and the SF-12 reflects perceived health and functioning over a broader time frame, approximately the previous month. These differences in temporal framing were considered when interpreting the observed associations.

### 2.4. Statistical Analysis

Pearson’s correlation coefficient was used to examine the linear associations between the BSRS total score and the PHQ-9 total score, between the BSRS total score and the SF-12 total score, and between the BSRS total score and the physical and mental health subscale scores of the SF-12. In addition, correlations between each BSRS item and the PHQ-9 total score were examined to further characterize the pattern of associations across questionnaire components.

A multiple linear regression model was then fitted to evaluate the simultaneous association of social rhythm dysregulation and depressive symptoms with perceived health-related quality of life. The SF-12 total score was entered as the dependent variable, whereas BSRS and PHQ-9 total scores were entered as the independent variables.

Regression coefficients are reported as model estimates derived from linear regression analysis. Given the exploratory nature of this secondary cross-sectional study, the model was intended to assess the main associations of interest rather than to provide a fully adjusted explanatory framework. Accordingly, results should be interpreted as indicative of associations. Accordingly, these analyses were not considered the primary basis for the study’s main inferential conclusions, which relied mainly on the scale-level associations and regression results. All statistical analyses were performed using IBM SPSS Statistics, version 24.

### 2.5. Missing Data Handling

In the parent trial protocol, missing data during follow-up were planned to be handled using last-observation-carried-forward, with multiple imputation for sensitivity analyses. Because the present study was restricted to baseline cross-sectional data, this longitudinal approach was not directly applicable. The analytical sample therefore consisted of participants with available baseline data on the variables examined in the current analysis.

### 2.6. Ethical Considerations

The survey was conducted in accordance with the Declaration of Helsinki and its revisions [43,44]. The Committee for Medical and Health Research Ethics of the Autonomous Region of Sardinia approved the study (reference number PG/2018/15546; 25 October 2018). Written informed consent was obtained from all participants.

## 3. Results

The sample consisted of 119 people, of whom 53 were male (44.6%) and 66 were female (55.4%); the mean age was 72.26 (±4.72 standard deviation).

As shown in Table 1, higher BSRS total scores were significantly associated with greater depressive symptom burden (r = 0.4106, *p* < 0.001). All individual BSRS items were also positively correlated with PHQ-9 scores, although the strength of the associations varied across routine domains.

Table 2 shows that greater social rhythm dysregulation was significantly associated with poorer perceived quality of life, both for the overall SF-12 score and for its physical and mental component summaries. In all cases, higher BSRS scores were related to lower SF-12 values.

Exploratory item-level correlations were also examined to describe the pattern of associations between questionnaire components. These analyses were considered descriptive and were interpreted with caution.

A multiple linear regression model was fitted with the SF-12 total score as the dependent variable and BSRS and PHQ-9 total scores as the independent variables. Both predictors were significantly associated with SF-12 scores. The overall model was statistically significant, with modest explanatory power (R^2^ = 0.148). Given the exploratory nature of the analysis, these results should be interpreted as indicative of associations rather than as a comprehensive or fully adjusted explanatory model.

## 4. Discussion

Our findings indicate that, in a sample of community-dwelling older adults aged 65 years or older and functionally able to engage in moderate physical activity, greater dysregulation of personal and social rhythms was associated with more depressive symptoms and poorer perceived quality of life. Multiple regression analysis further showed that both social rhythm dysregulation and depressive symptoms were significantly associated with quality of life. However, the regression model explained only a modest proportion of the variance in perceived quality of life. Therefore, although both variables were significantly associated with SF-12 scores, these findings should be interpreted as reflecting limited explanatory power rather than as evidence of a comprehensive model of quality of life in older adults. These results are in line with previous observations suggesting that instability in social rhythms may be related to reduced emotional resilience and a higher risk of depressive symptoms in older adults [4,5].

It is also important to note that the regression analysis was conducted within an exploratory framework and was not intended to provide a fully specified or diagnostically exhaustive model. Accordingly, the statistical reporting focused on the main coefficients and overall model fit, and more detailed reporting of precision estimates and diagnostic metrics was beyond the scope of the present secondary analysis. This aspect should be considered when interpreting the robustness and generalizability of the findings.

Another important consideration is that the regression model was not adjusted for a broader set of clinical and sociodemographic variables that are known to influence quality of life in older adults. Therefore, the observed associations may be affected by residual confounding. The model was intentionally specified in a parsimonious manner to explore the primary relationships of interest, but it should not be interpreted as a comprehensive or fully explanatory model of quality of life. Future studies with larger samples and more extensive covariate adjustment are needed to better disentangle these relationships.

Although participants were community-dwelling and functionally autonomous, this does not necessarily imply a high perceived quality of life. Perceived health and well-being may be influenced by psychological burden, rhythm disruption, and contextual or age-related factors that are not fully captured by functional independence alone.

From a clinical perspective, the present findings support the relevance of considering social rhythm regularity alongside depressive symptoms when examining well-being in later life. Within a broader chronobiological framework, regularity in sleep, meals, activity, and social contact may represent a meaningful behavioral correlate of perceived quality of life in older adults [2,3,10,11,13]. However, because of the cross-sectional design and the modest explanatory power of the regression model, these findings should not be interpreted as evidence of causality or of a fully explanatory model.

The broader conceptual frameworks discussed in the Introduction should be interpreted as contextual perspectives rather than as models directly tested in the present study, which was limited to the examination of cross-sectional associations among the measured variables.

The present results may also have practical implications. Interventions aimed at promoting regular daily routines, including stable sleep–wake schedules, meal timing, physical activity, daylight exposure, and social engagement, may be relevant targets for future research in older adults. At the same time, these implications should be considered preliminary, since the present study did not directly test intervention effects.

Strengths of the study include its focus on community-dwelling older adults, a population often underrepresented in this area of research, and the use of a multivariable approach to examine the associations of social rhythm dysregulation and depressive symptoms with quality of life [13,45]. Several limitations should also be acknowledged. The cross-sectional design precludes causal inference. All measures were self-reported, and no objective rhythm-related measures, such as actigraphy or light exposure assessment, were available [2,3,10]. Moreover, the regression model was not adjusted for a broader set of potential confounders relevant to older adults, and the explained variance was modest. Participants were community-dwelling, urban older adults enrolled in an active ageing program and medically able to engage in moderate physical activity, representing a relatively functional subgroup. Therefore, the generalizability of the findings to frailer, institutionalized, or more medically complex older populations may be limited [46].

In interpreting the present findings, it is important to consider that the instruments used capture partially different temporal frames (i.e., the BSRS refers to the previous week, the PHQ-9 to the previous two weeks, and the SF-12 to approximately the previous month). This temporal heterogeneity may have influenced the observed associations and further supports the need for a cautious interpretation of the results as reflecting cross-sectional relationships rather than temporally aligned processes [47,48,49,50,51,52,53,54,55,56].

Future studies should use longitudinal designs to clarify the temporal relationships among social rhythm dysregulation, depressive symptoms, and quality of life in older adults [57,58,59,60,61,62,63]. Research using objective rhythm-related measures and broader adjustment for relevant clinical and sociodemographic variables would help strengthen the interpretation of these associations. Intervention studies may also be useful to determine whether rhythm-focused strategies can improve well-being and quality of life in later life [64,65,66,67,68,69].

Overall, the present findings support the relevance of social rhythm dysregulation as a clinically meaningful correlate of well-being in later life. However, given the cross-sectional nature of the study and the modest explanatory power of the model, the results should be interpreted cautiously and as associative rather than causal [70,71,72,73,74].

## 5. Conclusions

In this sample of community-dwelling older adults (mean age, 72.3 years), greater dysregulation of social rhythms, including sleep, eating, and social contact patterns, as well as more depressive symptoms, were associated with a poorer perceived quality of life. Greater rhythm dysregulation was related to higher depressive symptom burden and lower quality of life, and both BSRS and PHQ-9 scores were significantly associated with SF-12 scores in the regression model (BSRS: β = −0.1863, *p* < 0.001; PHQ-9: β = −0.2885, *p* = 0.037; model R^2^ = 0.148). However, the explained variance of the model was modest, indicating that these variables accounted for a limited proportion of the variability in perceived quality of life. Within the broader framework of rhythmicity in psychiatry and the DYMERS perspective, these findings support the relevance of disruption in daily routines as a clinically meaningful correlate of poorer well-being in later life.

These findings should be interpreted within the context of a relatively functional sample of community-dwelling older adults and may not be directly generalizable to more vulnerable or clinically complex populations.

From a clinical and public health perspective, assessing social rhythms alongside depressive symptoms may help identify older adults at greater risk of reduced quality of life. Interventions aimed at promoting regular daily routines, including stable sleep–wake schedules, consistent meal timing, physical activity, appropriate light exposure, and social engagement, may represent relevant targets for future research in healthy aging. In this context, quality of life may be a useful outcome for evaluating the broader impact of rhythm-oriented approaches in older adults.

## Figures and Tables

**Table 1:** Correlation between BSRS and PHQ-9 in the examined sample.

Score	R	*p*	Mean ± SD
PHQ-9 score	-	-	2.31 ± 3.04
BSRS score	0.4106	<0.001	19.21 ± 6.90
BSRS Item 1: “Go To Sleep From Monday To Friday”	0.3063	<0.001	1.93 ± 0.99
BSRS Item 2: “Go To Sleep On The Weekend.”	0.2195	0.016	2.18 ± 1.15
BSRS Item 3: “Wake Up And Get Out Of Bed From Monday To Friday.”	0.3901	<0.001	1.89 ± 0.94
BSRS Item 4: “Wake Up And Get Out Of Bed On The Weekend.”	0.2765	0.002	1.97 ± 1.02
BSRS Item 5: “Meet Other People At School, At Work, Or During Commitments From Monday To Friday”	0.2919	0.001	2.01 ± 1.09
BSRS Item 6: “Meet Other People At School, At Work, Or During Commitments On The Weekend.”	0.2404	0.008	2.14 ± 1.08
BSRS Item 7: “Meet Other People In Your Free Time From Monday To Friday”	0.3776	<0.001	2.17 ± 1.16
BSRS Item 8: “ Meet Other People In Your Free Time On The Weekend.”	0.3659	<0.001	2.15 ± 1.15
BSRS Item 9: “Eat Main Meals From Monday To Friday”	0.2854	0.001	1.52 ± 0.59
BSRS Item 10: “Eat Main Meals On The Weekend”	0.2838	0.001	1.74 ± 0.84

Abbreviations: SD, standard deviation; BSRS, Brief Social Rhythm Scale; PHQ-9, Patient Health Questionnaire-9.

**Table 2:** Correlation between BSRS and SF-12 scores in the examined sample.

Score	R	*p*	Mean ± SD
BSRS Score	-	-	19.21 ± 6.90
SF-12 total score	−0.3974	<0.001	34.91 ± 5.09
SF-12 Physical Component Summary (PCS)	−0.3043	<0.001	14.11 ± 2.13
SF-12 Mental Component Summary (MCS)	−0.3619	<0.001	20.79 ± 3.79

Abbreviations: SD, standard deviation; BSRS, Brief Social Rhythm Scale; SF-12, Short Form-12 Health Survey.

## Data Availability

The data presented in this study are available upon request from the corresponding authors. Due to privacy and ethical issues, data are not publicly available.
